# Exploring causal correlations between inflammatory cytokines and ankylosing spondylitis: a bidirectional mendelian-randomization study

**DOI:** 10.3389/fimmu.2023.1285106

**Published:** 2023-11-20

**Authors:** Peng Fang, Xiaozhou Liu, Yang Qiu, Yang Wang, Dongsheng Wang, Jianning Zhao, Hao Ding, Nirong Bao

**Affiliations:** Department of Orthopedics, Jinling Hospital, Affiliated Hospital of Medical School, Nanjing University, Nanjing, China

**Keywords:** ankylosing spondylitis, inflammatory factors, mendelian randomization, GWAS, single nucleotide polymorphisms

## Abstract

**Background:**

The impact of inflammatory factors on the development of Ankylosing Spondylitis (AS) is widely recognized, but the exact causal relationship remains unclear.

**Methods:**

The bidirectional mendelian-randomization study utilized genetic data from a genome-wide association study (GWAS) of 186 AS cases and 456,162 controls of European ancestry. Inflammatory cytokines were obtained from a GWAS summary of 8,293 healthy participants. Causal associations were primarily investigated using the inverse variance-weighted method, supplemented by MR Egger, weighted median and weighted mode analyses. Heterogeneity in the results was assessed using the Cochrane Q test. Horizontal pleiotropy was evaluated through the MR-Egger intercept test and the MR pleiotropy residual sum and outliers (MR-PRESSO) test. Sensitivity analysis was conducted through leave-one-out analysis.

**Results:**

The results suggest a genetically predicted potential association between beta-nerve growth factor (βNGF), Interleukin-1-beta (IL-1β), and TNF-related apoptosis inducing ligand (TRAIL) with the risk of AS (OR: 2.17, 95% CI: 1.13-4.16; OR: 0.41, 95% CI: 0.18-0.95,; OR: 1.47, 95% CI: 1.02-2.13).Additionally, Interleukin-12p70 (IL-12p70), Interleukin-17 (IL-17), Interleukin-6 (IL-6), Interleukin-4 (IL-4), Stromal-cell-derived factor 1 alpha (SDF−1α), Macrophage inflammatory protein 1β (MIP1β), Monocyte chemoattractant protein-3 (MCP-3), Platelet-derived growth factor bb (PDGFbb), Granulocyte-colony stimulating factor (GCSF), Fibroblast growth factor basic (bFGF), TNF-related apoptosis inducing ligand (TRAIL), and Interferon-gamma (IFN -γ) are suggested as consequences of AS in genetically prediction.No evidence of horizontal pleiotropy or heterogeneity between the genetic variants was found (P>0.05), and a leave-one-out test confirmed the stability and robustness of this association.

**Conclusion:**

These findings suggest that βNGF, IL-1β, and TRAIL may play a crucial role in the pathogenesis of AS. Additionally, AS may impact the expression of cytokines such as IL-12p70, IL-17, IL-6, IL-4, SDF−1α, MIP1β, MCP-3, PDGFbb,GCSF, bFGF,TRAIL,and IFN-γ. Further investigations are warranted to determine whether these biomarkers can be utilized for the prevention or treatment of AS.

## 1 Introduction

AS is a chronic, progressive inflammatory joint disease that primarily affects the spine and pelvis. It is characterized by inflammation, stiffness, pain, and functional impairment of the spinal and pelvic joints (1). According to a global epidemiological study, the worldwide prevalence of AS is estimated to be between 0.1% and 1.4%. The variation within this range may be influenced by factors such as geography, ethnicity, and environment (2, 3).

At present, the precise mechanisms underlying AS remain somewhat elusive, but research has shed light on the intricate interplay between AS and inflammatory factors (4). In AS patients, the inflammatory response is often accompanied by aberrant production of multiple cytokines. For example, tumour necrosis factor-alpha (TNFα), interleukin-1 beta (IL-1β) and interleukin-17 (IL-17).Such irregular production and activation of these inflammatory factors can lead to joint and spinal inflammation, pain and tissue damage (5–7). Therapeutic approaches targeting these inflammatory factors, such as the use of biological agents to inhibit TNFα, have gained widespread acceptance in the treatment of AS (8). However, the debate as to whether inflammatory factors are the cause of AS or a consequence of its progression remains controversial. Although observational studies have attempted to elucidate the causal relationship between inflammatory factors and AS, the results may be susceptible to bias due to unanticipated confounders or reverse causality, making it difficult to establish a definitive causal relationship (9).

The Mendelian randomization study method serves as a powerful tool in epidemiological research, utilizing genetic variation as an instrument to assess the causal association between risk factors and specific diseases (10). In Mendelian randomization studies, genetic variation adheres to the principle of random allele allocation to offspring, akin to randomized controlled experiments. This approach effectively mitigates confounding factors and reverse causation often encountered in observational studies (11). Despite the widespread use of Mendelian randomization in exploring risk factors, no studies employing this approach have yet investigated the causal relationship between inflammatory factors and AS. Hence, the objective of this study was to employ a bidirectional two-sample Mendelian randomization study, aiming to unravel the potential causal link between inflammatory factors and AS, with the aim of providing strategies for the prevention and treatment of AS.

## 2 Materials and methods

### 2.1 Data sources and study design

**Figure 1:**
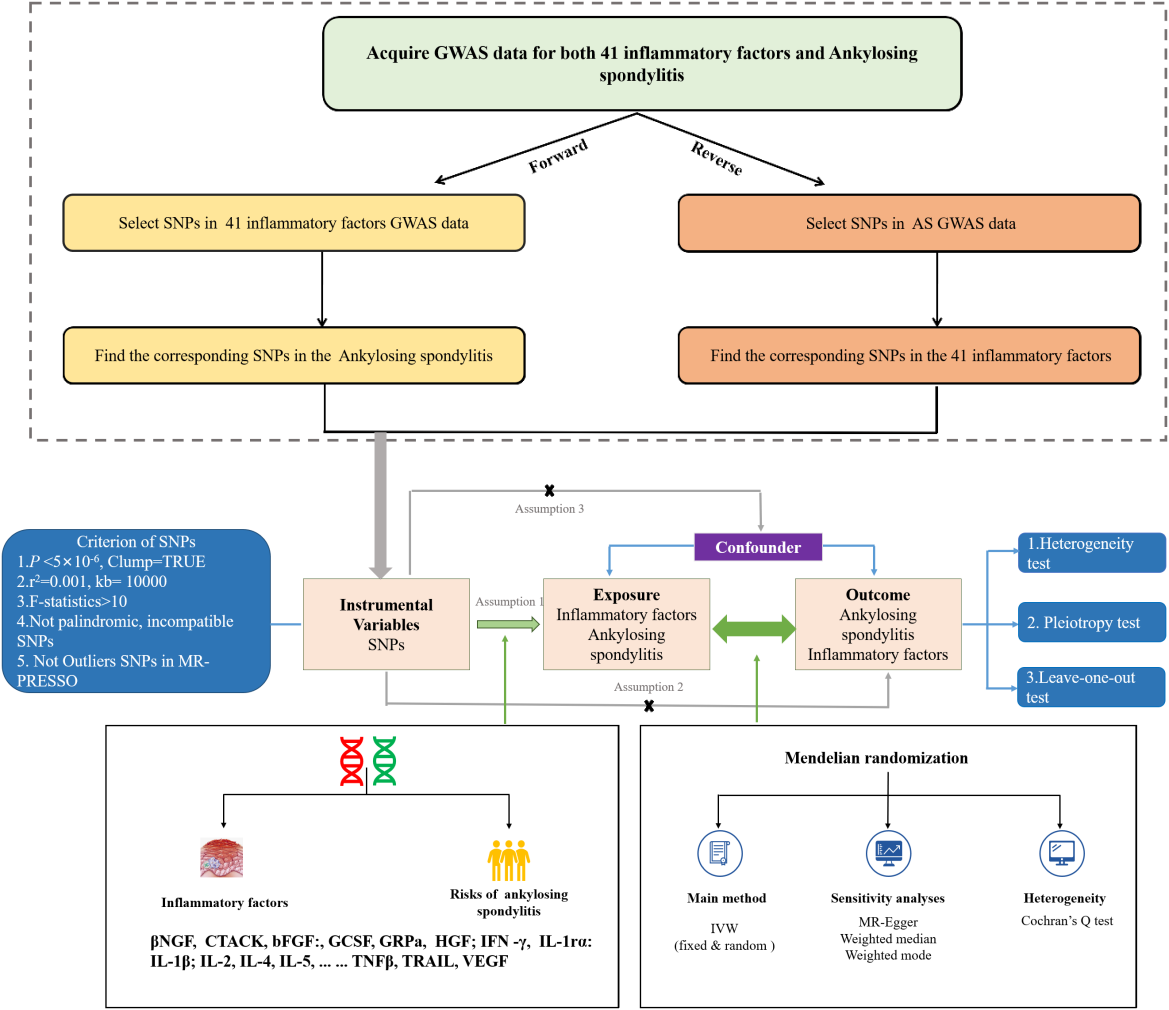
The flowchart of the Mendelian randomization study that the causal association between 41 inflammatory factors and AS.

This study uses large-scale GWAS summary datasets where all participants gave informed consent in their respective original studies. As we only rely on summary level statistics, no additional ethical approval is required. The GWAS data utilized in the MR analysis for 41 inflammatory factors were sourced from 8293 Finns individuals, included 41 inflammatory factors (12). GWAS data for these 41 inflammatory factors can also be found in the IEU Open GWAS project (https://gwas.mrcieu.ac.uk/) with GWAS IDs, as indicated in **Supplementary 1**.Summary statistics for AS were obtained from the UK Biobank, which included 186 cases and 456162 controls of European ancestry, using a generalized linear mixed model (GLMM)-based method called (fast GWA-GLMM) with adjustment for covariates (13–15) We performed a bidirectional two-sample MR study to assess the causality of inflammatory factors and AS, using single nucleotide polymorphisms (SNPs) as instrumental variables (IVs).These SNPs had to meet three assumptions: assumption 1: these SNPs must be strongly correlated with exposure; assumption 2: these SNPs effect on results only through exposure; assumption 3: these SNPs are not related to confounding factors (16). **Figure 1** illustrates the flow chart of the entire analysis.

### 2.2 Selection of instrumental variables

Initially, we established a genome-wide significant threshold of *P*<5×10^-8^ to identify highly correlated SNPs with inflammatory cytokines and AS. However, due to the limited number of SNPs identified for certain inflammatory cytokines and AS when they were considered as the exposure, we opted for a slightly higher cutoff (*P*< 5×10^-6^) (17). To ensure the selection of independent SNPs and minimize the impact of linkage disequilibrium (LD) on the results, we established a threshold of 0.001 for the linkage disequilibrium parameter (r^2^) and a genetic distance of 10,000kb (18). The strength of the correlation between instrumental variables and exposure factors was assessed using the F statistic. To mitigate bias resulting from weak instrumental variables, we only considered SNPs with an F statistic greater than 10 (19).

### 2.3 Statistical analysis

We performed a bidirectional two-sample Mendelian randomization (MR) study investigating the relationship between inflammatory cytokines and AS using the “TwoSampleMR” package in R software (version 4.1.2). The MR analysis employed various methods, including the random-effects variance-weighted model (IVW), MR-Egger, weighted median, and weighted mode. The random-effects IVW served as the primary method, while MR-Egger, weighted median, and weighted mode were supplementary methods employed to ensure the robustness of the results (20). To assess the heterogeneity of SNP effects associated with inflammatory cytokines and AS, we utilized the I2 index and Cochran’s Q statistic for MR-IVW analyses, and Rucker’s Q statistic for MR-Egger analyses. A p-value greater than 0.05 indicated no significant heterogeneity (21). Additionally, we employed both the MR-Egger method and the MR pleiotropy residual sum and outlier (MR-PRESSO) method to test for horizontal pleiotropy (22). Furthermore, a ‘leave one out’ analysis was conducted to examine whether the causal relationship between exposure and outcome was influenced by a single SNP. A p-value greater than 0.05 indicated no evidence of horizontal pleiotropy (23). To account for multiple testing, we applied the Bonferroni method, which led us to consider associations with P-values below 0.0012 (0.05/41) as strong evidence of associations. Results with P-values ranging from 0.0012 to 0.05 were considered suggestive associations (24).

## 3 Results

### 3.1 Influence of 41 inflammatory cytokines on AS

**Figure 2:**
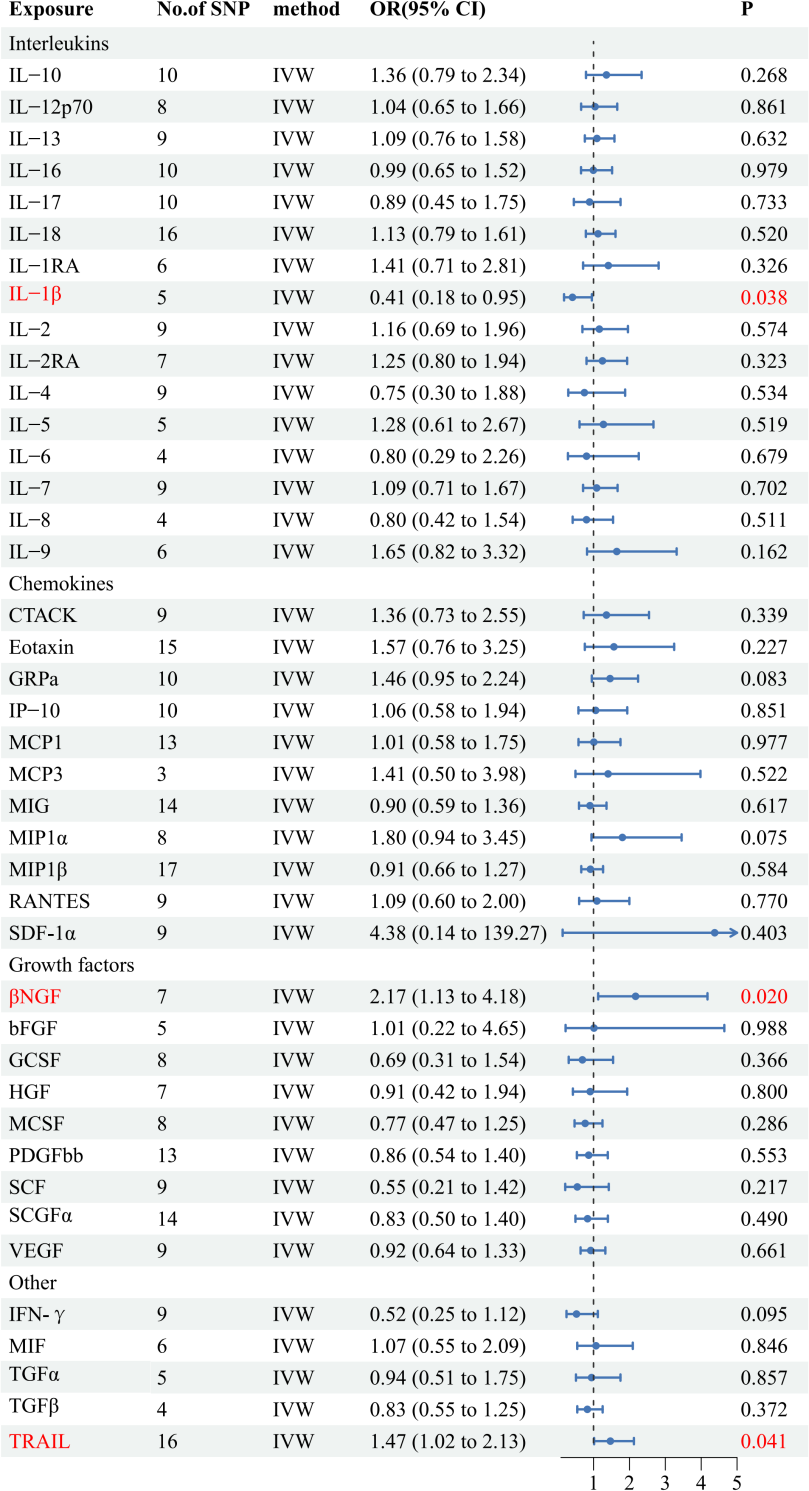
Forest plots of the causal relationship between 41 inflammatory factors and AS in the result of IVW in the forward MR analysis.

**Figure 3:**
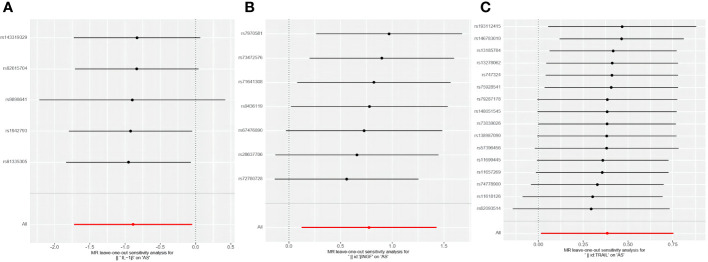
Forest plots of causal relationship between 3 inflammatory factors—IL-1β **(A)**, βNGF **(B)**, and TRAIL **(C)** and AS in the results of 'leave-one-out' analysis in the forward analysis.

Among the accessible inflammatory factors, Nine out of forty-one exhibited three or more valid SNPs when the genome-wide significance cutoff was set at 5× 10^-8^. For the inflammatory factors, a higher threshold of *P*<5×10^-6^ was employed to ensure an adequate number of SNPs for subsequent MR analysis. Additionally, all F-statistic values exceeded 10, indicating minimal likelihood of weak instrument bias (**Supplementary 2**). To evaluate the influence of 41 inflammatory cytokines on AS, we employed IVW as the primary method, complemented by MR Egger, weighted median, and weighted mode. The IVW analysis revealed a significant positive correlation between βNGF and AS, with an odds ratio (OR) of 2.17 (95% CI=1.13-4.81, *P*=0.020). Similarly, TRAIL was also found to be positively associated with AS, with an OR of 1.47 (95% CI=1.02-2.13, *P*=0.041), whereas IL-1β exhibited a negative correlation with AS, with an OR of 0.41 (95% CI=0.18-0.95, *P*=0.038).The results of the IVW,MR Egger, weighted median, and weighted mode for the 41 inflammatory cytokines can be found in **Figure 2** and **Supplementary 3**. No evidence of heterogeneity was found in the Cochran Q-test for these three inflammatory factors in the MR analysis of AS (Q value = 5.911, *P*=0.433; Q value = 19.834, *P*=0.178; Q value=0.483, *P*=0.975). Additionally, no significant intercepts were observed (intercepts=-0.305, *P*=0.271; intercepts=-0.063, *P*=0.375; intercepts = 0.008,*P*=0.960), indicating the absence of pleiotropy. Similarly, the results of MR-PRESSO indicated no horizontal multidirectionality in this MR analysis (RSSobs =7.881,*P*=0.479; SSobs=22.298, *P*=0.219;SSobs=0.601,*P*= 0.986). **Table 1** provides a summary of the multidirectionality and heterogeneity tests. Furthermore, the “leave-one-out” analysis demonstrated the robustness of our MR analysis, as it was not influenced by any individual SNP (**Figure 3**).

**Table 1:** The results of heterogeneity and horizontal pleiotropy of the 3 inflammatory factors and AS in the Forward MR analysis.

Exposure	Outcome	Heterogeneity test		Pleiotropy test	MR-PRESSO	
		Cochran’s Q test	Rucker’s Q test	Egger intercept	Distortion	Global
		(*P* value)	(*P* value)	(*P* value)	test	Test
		IVW	MR-egger	MR-egger	Outliers	*P* Value
IL-1β	Ankylosing Spondylitis	0.975	0.923	0.960	NA	0.986
βNGF	Ankylosing Spondylitis	0.433	0.599	0.271	NA	0.479
TRAIL	Ankylosing Spondylitis	0.178	0.176	0.375	NA	0.986

### 3.2 Influence of AS on 41 inflammatory cytokines

**Figure 4:**
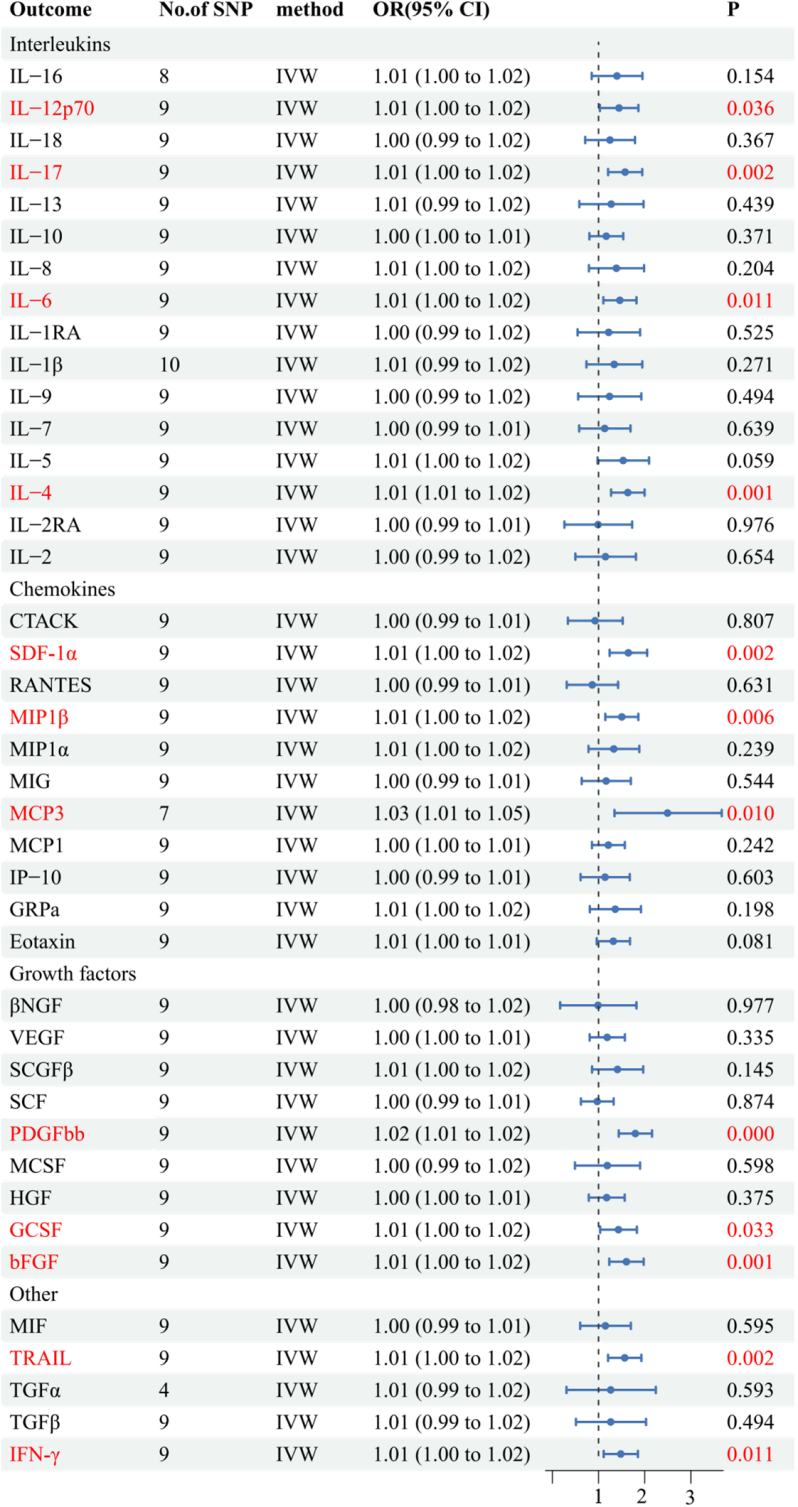
Forest plots of the causal relationship between AS and 41 inflammatory factors in the result of IVW in the reverse MR analysis.

**Figure 5:**
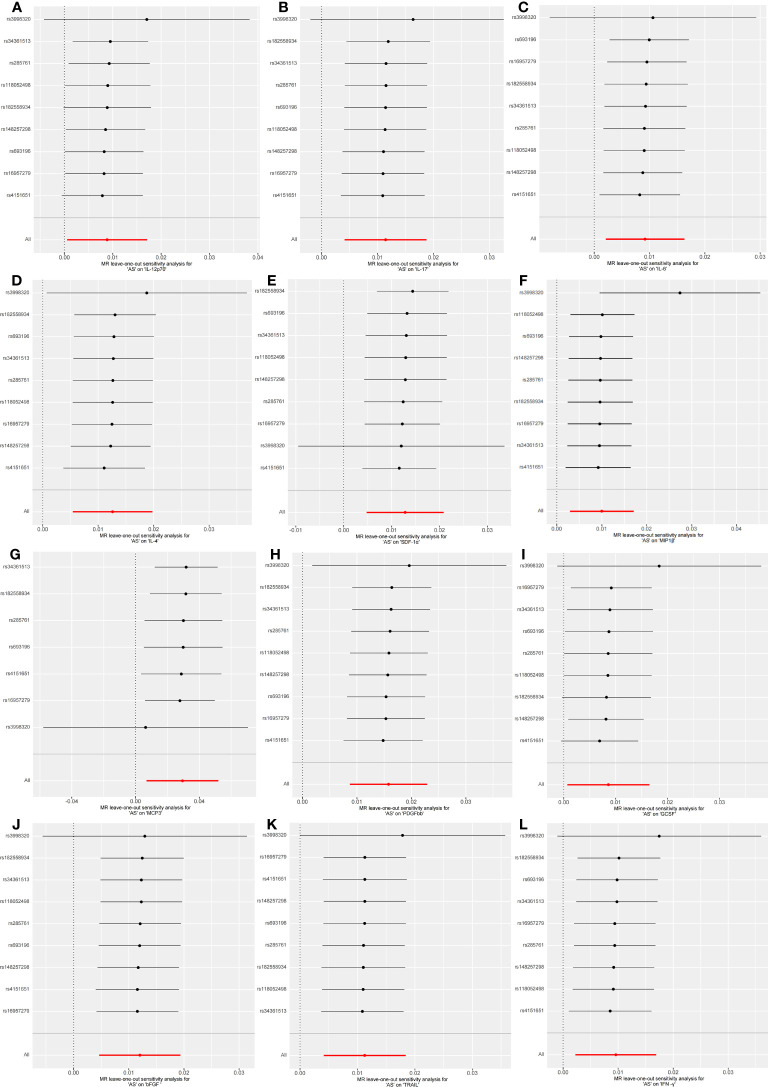
Forest plots of causal relationship between AS and 12 inflammatory factors IL-12p70 **(A)**, IL-17 **(B)**, IL-6 **(C)**, IL-4 **(D)**, SDF-1α **(E)**, MIP1β **(F)**, MCP3 **(G)**, PDGFbb **(H)**, GCSF **(I)**, bFGF **(J)**, TRAIL **(K)**, IFN-γ **(L)** in the results of 'leave-one-out' analysis in the reverse analysis.

Initially, when the genome-wide significance cutoff was set at 5 × 10^-8^, there were insufficient SNPs available for the MR analysis. Therefore, we adjusted the threshold to 5 × 10^-6^ to ensure an adequate number of SNPs for the analysis. Additionally, all F-statistic values exceeded 10, indicating minimal likelihood of weak instrument bias (**Supplementary 4**).The IVW results suggest that IL-12p70, IL-17, IL-6, IL-4, SDF−1α, MIP1β, MCP-3, PDGFbb, GCSF, bFGF, TRAIL, and IFN-γ may be consequences of AS (OR: 1.01, 95% CI: 1.00-1.02, P=0.036; OR: 1.01, 95% CI: 1.00-1.02, P=0.002; OR: 1.01, 95% CI: 1.00-1.02, P=0.011; OR: 1.01, 95% CI: 1.01-1.02, P=0.001; OR: 1.01, 95% CI: 1.00-1.02, P=0.002; OR: 1.01, 95% CI: 1.00-1.02, P=0.006; OR: 1.03, 95% CI: 1.01-1.05, P=0.010; OR: 1.02, 95% CI: 1.01-1.02, P=0.000; OR: 1.01, 95% CI: 1.00-1.02, P=0.033; OR: 1.01, 95% CI: 1.00-1.02, P=0.001; OR: 1.01, 95% CI: 1.00-1.02, P=0.002; OR: 1.01, 95% CI: 1.00-1.02, P=0.011). **Figure 4** and **Supplementary 5** provide the results of the IVW, MR Egger, weighted median, and weighted mode analysis. The Cochran Q test did not detect any evidence of heterogeneity, and there was no significant intercept observed, indicating the absence of pleiotropy. Additionally, the results of MR-PRESSO indicated no horizontal pleiotropy in this MR analysis. **Table 2** summarizes the results of the pleiotropy and heterogeneity tests. The “leave-one-out” analysis demonstrated the robustness of our MR analysis, as it was not influenced by any individual SNP (**Figure 5**).

**Table 2:** The result of heterogeneity and horizontal pleiotropy of the AS and 12 inflammatory factors in the reverse MR analysis.

Exposure	Outcome	Heterogeneity test		Pleiotropy test	MR-PRESSO	
		Cochran’s Q test	Rucker’s Q test	Egger intercept	Distortion	Global
		(*P* value)	(*P* value)	(*P* value)	test	Test
		IVW	MR-egger	MR-egger	Outliers	*P* Value
Ankylosing Spondylitis	IL-12p70	0.208	0.194	0.432	NA	0.429
Ankylosing Spondylitis	IL-17	0.875	0.840	0.574	NA	0.853
Ankylosing Spondylitis	IL-6	0.473	0.482	0.331	NA	0.710
Ankylosing Spondylitis	IL-4	0.664	0.558	0.948	NA	0.667
Ankylosing Spondylitis	SDF-1α	0.291	0.242	0.557	NA	0.566
Ankylosing Spondylitis	MIP1β	0.581	0.829	0.125	NA	0.479
Ankylosing Spondylitis	MCP3	0.242	0.160	0.939	NA	0.939
Ankylosing Spondylitis	PDGFbb	0.610	0.563	0.490	NA	0.745
Ankylosing Spondylitis	GCSF	0.303	0.239	0.652	NA	0.459
Ankylosing Spondylitis	bFGF	0.887	0.837	0.689	NA	0.943
Ankylosing Spondylitis	TRAIL	0.980	0.973	0.611	NA	0.868
Ankylosing Spondylitis	IFN -γ	0.553	0.468	0.661	NA	0.597

## 4 Discussion

To the best of our knowledge, this study represents the first comprehensive evaluation of the causal effects of 41 inflammatory factors on AS and vice versa. Using a bidirectional two-sample MR analysis with two independent populations, we have made significant findings. Our results indicate that genetically predicted bNGF and TRAIL are positively associated with the risk of AS, while IL-1b shows a negative association with AS risk. Additionally, we observed that AS is associated with increased levels of IL-12p70, IL-17, IL-6, IL-4, SDF-1α, MIP1β, MCP-3, PDGFbb, GCSF, bFGF, TRAIL, and IFN-γ. These findings were generally robust in sensitivity analysis. These findings provide valuable insights for the prevention and treatment of AS.

The pathogenesis of AS remains incompletely understood; however, it is widely believed that the pivotal role lies in the aberrant production and regulation of inflammatory factors (25). This dysregulation of inflammatory factors elicits an abnormal immune response in individuals with AS, subsequently triggering an inflammatory cascade. Primarily affecting the spinal and pelvic joints, this inflammatory response gives rise to arthritis and inflammatory damage to ligaments and bones. Consequently, individuals with AS experience symptoms including spinal stiffness, pain, and functional impairment (26). While these studies have shed light on the involvement of inflammatory factors in AS, they have yet to definitively establish whether these factors are the cause or the consequence of the condition.

Previous research has elucidated the pivotal role of inflammation in the pathogenesis of AS (27). Various inflammatory molecules, including IL-6, IL-17, and TNFα, have been proposed as potential serum biomarkers for AS (26). However, these observational studies are susceptible to confounding factors and reverse causation, which can distort the true cause-and-effect relationships. To address this, we conducted a bidirectional two-sample MR analysis to unravel the upstream and downstream regulators of inflammation in AS. Consistent with prior findings, our results affirm that heightened levels of βNGF and TRAIL are associated with an increased risk of AS. Notably, βNGF, a versatile neurotrophic factor, assumes a paramount role in the generation and perpetuation of deleterious and neuropathic pain (28). In the context of arthritis, elevated concentrations of nerve growth factor have been observed in synovial fluid (29). Thus, βNGF may potentially contribute to the pathogenesis of AS, although the underlying mechanisms remain to be elucidated. Tumour necrosis factor-related apoptosis-inducing ligand (TRAIL), a member of the tumour necrosis factor (TNF) superfamily, plays a significant role in the pathogenesis of autoimmune diseases (30). A study unveiled a substantial increase in serum TRAIL-R1 levels among patients with AS compared to the control group, with values of 4.5 ± 2.3 pg/mL and 3.5 ± 2.3 pg/mL, respectively (p = 0.036) (31). Moreover, our findings suggest a reciprocal causation between TRAIL and AS. In AS, IL-1β is also considered a critical pro-inflammatory cytokine, and its expression levels may be upregulated, similar to findings by Zambrano-Zaragoza and Peng (32, 33), who observed elevated IL-1β levels in AS patients. However, this does not necessarily imply that IL-1β can cause AS; it could also be a negative feedback regulation by the body to protect itself. Additionally, Peng also found that the levels of IL-1β did not significantly change in AS patients before and after receiving tumor necrosis factor inhibitors. This suggests that while IL-1β may be related to the pathogenesis of AS, it is not directly influenced by tumor necrosis factor inhibitors. Other factors or pathways may contribute to the increased levels of IL-1β in AS patients. Noteworthy, Chinese researchers observed a notable rise in the average plasma IL-1β concentration among patients with AS in comparison to the control group. Additionally, they identified the IL-1β rs2853550 AG genotype as a genetic factor contributing to the risk of developing AS in the Chinese population (34). They also discovered a negative correlation between IL-1β SNPs, rs3783550 and rs3783546, and AS. However, some studies have reported no elevation in serum IL-1β levels among patients with AS (35). Furthermore, Wu reported a meta-analysis indicating that the allelic variant +889 of IL-1α gene (rs1800587) increased the risk of AS in European populations OR=1.357, 95%CI=1.08–1.697, *P*=0.007), while no association was found between the AS and the two IL-1β SNPs, -511 and +3953, in the same population (36). Our conclusion is based on the results of a meta-analysis of five IL-1β SNPs. These discrepancies may arise from factors such as small sample sizes, low statistical power, clinical heterogeneity, and racial differences (37). In the future, further research will be needed to clarify the causal relationship between IL-1β and AS.

Our MR results showed that IL-12p70, IL-17, IL-6, IL-4, SDF-1α, MIP1β, MCP-3, PDGFbb, GCSF, bFGF, TRAIL, and IFN-γ, may play key roles as AS downstream factors. A review of the relevant literature also seems to lead to a relevant theoretical basis. Saliva samples from individuals with AS exhibit elevated levels of IL-12p70. The differentiation of Th1 cells induced by IL-12p70 may potentially contribute to the development of organ-specific autoimmune diseases (38). IL-17 is the major cytokine in AS, and studies have shown that serum levels of IL-17 are higher in patients with AS (39). In addition, it has been shown that AS can be greatly ameliorated by blocking the IL-23/IL-17 pathway, which further suggests that IL-17 plays an important role in the development of AS (40). IL-6 is the second most important inflammatory cytokine in rheumatic diseases, and levels of IL-6 are higher in patients with AS than in controls, especially in the early stages of the disease, in serum, cartilage, synovial fluid and sacroiliac joint biopsy specimens (41). IL-6-induced STAT 3 phosphorylation contributes to increased pTh17 responses in AS peripheral arthritis patients (42). These molecular mechanisms provide strong support for our MR results. The relationship between IL-4 and AS has not been reported, but IL-4 plays an important role in in inflammatory arthritis and enthesitis (43, 44). IL-4 has anti-inflammatory effects, and our MR results indicate a positive correlation between AS and IL-4, which could be a form of negative feedback regulation in the body for self-protection. However, this is only a hypothesis and will require further validation in the future.SDF-1α, also known as CXCL12, is a key contributor to pathological new bone formation in AS (45). Macrophage inflammatory protein-1β(MIP-1β/CCL4) is an essential chemotactic cytokine in the immune response against infection and inflammation; it attracts other cells to the local area to exert its biological effects. Few observational clinical studies or meta-analyses have found MIP-1β to be associated with AS, but our current MR analyses determined that AS may lead to elevated levels of MIP-1β.There is a scarcity of studies investigating the role of MCP-3, GCSF, and bFGF in the progression of AS. While we have been observed that AS can result in increased levels of these inflammatory factors, the underlying mechanisms are yet to be fully understood. Research has shown that PDGF-BB levels in AS patients are higher than those in the normal control group (46). PDGF-BB)/PDGFR-β pathway is generally considered an important pathway for promoting bone formation. PDGF-BB, as a growth factor derived from platelets, is regarded as a key factor in the pathological bone formation of AS (47, 48). The relationship between IFN-γ and AS has been extensively studied. IFN-γ is a cytokine produced mainly by activated T cells and natural killer cells, and is involved in immune regulation and inflammatory responses. Studies have shown that the levels of IFN-γ are significantly elevated in the blood and joint tissues of patients with AS. High levels of IFNG are closely associated with the inflammatory response and disease progression in AS (49).

The present study used a two-way Mendelian randomization approach to investigate the causal relationship between inflammatory factors and AS, and explored the intrinsic link between the two through a review of the relevant literature. The greatest advantage of this study over traditional observational studies is that causality estimation avoids reverse causality and confounding bias. However, some limitations of this study should not be overlooked. Firstly, all participants in the GWAS were of European ancestry, and therefore, the generalizability of our findings to other populations and regions remains to be determined. Secondly, in the GWAS data for inflammatory cytokines and AS, we used a significance cut-off of *P*-value < 5 × 10^-6^ because insufficient SNPs available for the MR analysis at a cut-off of *P*-value <5×10^-8^,which was considered as rational threshold (50). Finally no causal relationship was found between TNFα and TNFβ and AS. this may be related to the relatively small number of SNPs included in the Mendelian randomization study, and in the future, more SNPs or an expanded sample size may be needed to further investigate this issue. Nevertheless, this study may be the first to utilize the Mendelian randomization research method to explore the causal relationship between 41 inflammatory factors and AS, providing valuable insights for this field.

## 5 Conclusion

We employed a bidirectional Mendelian randomization approach using two independent samples to investigate the causal relationship between inflammatory factors and AS. We identified the upstream and downstream regulatory factors of inflammatory cytokines in AS. Based on our research findings, we draw the following conclusions: βNGF, IL-1β, and TRAIL are considered upstream factors in the pathogenesis of AS, while IL-12p70, IL-17, IL-6, IL-4, SDF-1α, MIP1β, MCP-3, PDGFbb, GCSF, bFGF, TRAIL, and IFN-γ may play key roles as downstream factors in AS. These inflammatory factors play significant roles in the pathogenesis of AS. Although the specific mechanisms of some inflammatory factors have not been fully elucidated, this study provides relevant clues, especially concerning IL-4 and PDGF-BB. Further research will contribute to a deeper understanding of the specific roles of these inflammatory factors in the development of AS and provide new targeted strategies for the prevention and treatment of AS.

## Data availability statement

The original contributions presented in the study are included in the article/**Supplementary Material**. Further inquiries can be directed to the corresponding authors.

## Ethics statement

Ethical approval was not required for the study involving humans in accordance with the local legislation and institutional requirements. Written informed consent to participate in this study was not required from the participants or the participants’ legal guardians/next of kin in accordance with the national legislation and the institutional requirements.

## Author contributions

PF: Data curation, Software, Visualization, Writing – original draft, Writing – review & editing. XL: Funding acquisition, Writing – review & editing. YQ: Conceptualization, Data curation, Methodology, Writing – review & editing. YW: Formal analysis, Investigation, Software, Supervision, Writing – review & editing. DW: Funding acquisition, Methodology, Project administration, Resources, Writing – review & editing. JZ: Data curation, Formal analysis, Methodology, Project administration, Resources, Supervision, Validation, Visualization, Writing – review & editing. HD: Conceptualization, Data curation, Formal analysis, Investigation, Methodology, Software, Supervision, Visualization, Writing – review & editing. NB: Conceptualization, Data curation, Formal analysis, Investigation, Methodology, Project administration, Software, Visualization, Writing – review & editing.
